# Supplementary material for: Microbial mat compositions and localization patterns explain the virulence of black band disease in corals
Source: NPJ Biofilms Microbiomes. 2023 Apr 4;9:15. doi: 10.1038/s41522-023-00381-9 (PMC10073141; doi:10.1038/s41522-023-00381-9)
Supplement: Supplementary file 1 — Supplementary Materials [file 41522_2023_381_MOESM1_ESM.pdf]

Supplementary materials for

Microbial mat compositions and localization patterns explain the virulence of black band disease in corals.

Naohisa Wada, Akira Iguchi, Yuta Urabe, Yuki Yoshioka, Natsumi Abe, Kazuki Takase, Shuji Hayashi, Sakiko Kawanabe, Yui Sato, Sen-Lin Tang*, Nobuhiro Mano*

* These authors jointly supervised this work

Supplementary Tables	Page 2 - 7
Supplementary Figures	Page 8 - 14
Supplementary Note	Page 15 - 16
Supplementary References	Page 16 - 17

Supplementary Tables

Supplementary Table 1 Number of OTUs and the relative abundance at total sum scaling in representative bacterial families.

	Family	Number of OTUs	Average of total relative abundance (%)	Ranging among the samples (%)
Cyanobacteria	*Desertifilaceae*	7	42.57	1.39 – 77.68
	Oscillatoriaceae	3	1.10	0 – 9.75
Alfaproteobacteria	*Rhodobacteraceae*	44	5.64	0.04 – 26.03
Gammaproteobacteria	*Vibrionaceae*	12	4.01	0.15 – 18.79
	Alteromonadaceae	17	5.24	0.30 – 24.41
	*Alteromonadaceae*_unclassified	3	3.97	0.12 – 15.56
	Colwelliaceae	8	2.79	0.03 – 7.05
	Nitrincolaceae	7	2.23	0 – 13.19
	Pseudoalteromonadaceae	5	2.18	0.01 – 21.43
	Saccharospirillaceae	4	1.42	0.06 – 6.91
Deltaproteobacteria	*Desulfovibrionaceae*	8	3.14	0 – 13.41
	Desulfobacteraceae	7	2.52	0 – 8.72
	Desulfococcaceae	1	0.53	0 – 3.78
Campilobacterota	*Arcobacteraceae*	14	5.55	0.02 – 17.63
	Rs-M59_termite_group	1	0.51	0 – 6.11
Firmicutes	*Caminicellaceae*	1	1.20	0 – 3.99
	Lachnospiraceae	4	1.17	0 – 3.15
	*Lachnospirales*_unclassified	4	1.11	0 – 2.86
	Clostridiaceae	3	0.68	0 – 3.05
Bacteroidota	*Flavobacteriaceae*	67	1.06	0.05 – 5.79
	Saprospiraceae	76	0.83	0 – 4.00
	*Bacteroidia*_unclassified	49	0.53	0.02 – 1.72
Deferribacterota	*Deferribacteraceae*	1	0.61	0 – 3.78
Verrucomicrobiota	P.palmC41_fa	2	0.55	0 – 3.63
Others (< 0.5%)	–	1339	7.21	1.23 – 40.05

Supplementary Table 2 Summary of representative OTUs (showing > 1% at total sum scaling) in BBD with various linear-migration rates (n=12).

Family	OTU	The relative abundance ranged along the samples (%) *1	Taxonomy *2	Closest sequence(s) in phylogenetic position			
				Similarity (%)	GeneBank Acc. No.	Source	Reference
Desertifilaceae	OTU 1	1.38 – 77.67 (n=12)	*Roseofilum* AO1-A	100	NR_116573	BBD in the Red Sea	[1]
				100	KU579375.1	BBD in Australia	[2]
				100	MH341659.1	BBD in the Red Sea	[3]
				100	LC368145.1	BBD in Japan	[4]
Oscillatoriaceae	OTU 23	0.03 – 9.71 (n=3)	Uncultured bacteria	98.01	MT321585.1	Boat launch in USA	[5]
				96.02	DQ446127.2	BBD in Bahamas	[6]
Desulfovibrionaceae	OTU 12	0.01 – 4.10 (n=10)	*Desulfovibrio*	100	AY497300.1	BBD in Netherlands Antilles	[7]
Desulfobacteraceae	OTU 5	0.02 – 7.98 (n=10)	*Desulfocella*	100	GU319466.1	Coral treated in high pH from the Red Sea	[8]
				99.6	KC527305.1	Coral white plague disease in Thailand	[9]
Rhodobacteraceae	OTU 6	0.01 – 13.47 (n=11)	*Ruegeria*	100	MW589660.1	Sea cucumber from Mexico	Quintanilla-Mena et al 2021, Unpublished
				100	AF473938.1	BBD in US Virgin Islands	[10]
	OTU 24	0.01 – 6.96 (n=12)	*Thalassobius*	100	GU472129.1	BBD in the Red Sea	Arotsker et al. 2010, Unpublished
				100	MH341656.1	BBD in the Red Sea	[3]

Arcobacteraceae	OTU 8	0.54 – 10.84 (n=6)	Uncultured bacteria	100	MH341640.1	BBD in the Red Sea	[3]
				100	EF123613.1	BBD in US Virgin Islands	[6]
	OTU 9	0.03 – 10.81 (n=9)	*Arcobacteraceae*_unclassified	100	MH341647.1	BBD in the Red Sea	[3]
				100	HM768558.1	BBD in Netherlands Antilles	[11]
	OTU 11	0.01 – 5.46 (n=12)	*Arcobacteraceae*_unclassified	98.88	MH341652.1	BBD in the Red Sea	[3]
				98.88	LT904749.1	–	Latif-Eugenin et al. 2017, Unpublished
Alteromonadaceae	OTU 3	0.26 – 20.28 (n=12)	*Alteromonas*	100	LR812090.1	–	Duchaud 2020, Unpublished
Vibrionaceae	OTU 7	0.01 – 18.74 (n=12)	*Vibrionaceae*_unclassified	99.60	CP045350.1	Tank containing coral in Germany	Rockert et al 2019, Unpublished
				99.60	NW828438.1	Coral in Australia	Kuek et al. 2021, Unpublished
				99.60	NW872696.1	Coral	Loughran et al 2021, Unpublished
*Alteromonadales*_unclassified	OTU 2	0.12 – 15.56 (n=12)	*Alteromonadales*_unclassified	99.60	FJ202858.1	Coral white plague in Puerto Rico	[12]
Colwelliaceae	OTU 4	0.02 – 6.90 (n=12)	*Thalassotalea*	100	HM768682.1	BBD in Netherlands Antilles	[11]
				100	KC527285.1	Coral white plague disease in Thailand	[9]
Nitrincolaceae	OTU 14	1.13 – 13.19 (n=2)	*Marinobacterium*	100	MH341660.1	BBD in the Red Sea	[3]
				100	FJ202983.1	Coral white plague in Puerto Rico	[12]

Pseudoalteromonadaceae	OTU 10	0.01 – 15.86 (n=11)	*Algicola*	100	FJ202088.1	Coral white plague in Puerto Rico	[12]
Saccharospirillaceae	OTU 19	0.01 – 5.14 (n=11)	*Thalassolituus*	99.60	HQ317342.1	Biofilm of coral reef in Indonesia	Catalano er al 2010, Unpublished
				99.60	MF039943.1	Sponge	Keren et al 2010, Unpublished
Caminicellaceae	OTU 13	0.08 – 3.99 (n=9)	*Paramaledivibacter*	100	AY148309.1	BBD in Australia	Cooney and Bythell 2002, Unpublished
				100	AY348731.1	Healthy tissue in coral disease from Australia	[13]
Lachnospiraceae	OTU 15	0.26 – 3.15 (n=9)	*Lachnospiraceae*_unclassified	100	MH341658.1	BBD in Red Sea	[3]
				100	HM768582.1	BBD in Netherlands Antilles	[11]
*Lachnospirales*_unclassified	OTU 16	0.47 – 2.78 (n=9)	Lachnospirales_unclassified	100	GU471984.1	BBD in Red Sea	Arotsker et al, 2020, Unpublished
				100	GQ455295.1	BBD in Red Sea	[14]

*1 The proportions represent only the relative abundance of > 0.01% at total sum scaling found in each sample.

*2 The Taxonomy defined base on Silva SSU ref v138.

Supplementary Table 3 Partial correlation between the clr-transformed abundance of representative bacterial families and liner-migration rates. The clr-transformed abundance matrix was summed across the OTUs belonging to the corresponding family.

		Spearman's rank correlation		
	Representative family	s	Correlation coefficient (ρ)	*p* value*[1]
Cyanobacteria	*Desertifilaceae*	130	0.5454545	0.07068
	Oscillatoriaceae	338	–0.1818182	0.573
Alfaproteobacteria	*Rhodobacteraceae*	456	–0.5944056	**0.04575***
Gammaproteobacteria	*Vibrionaceae*	224	0.2167832	0.4991
	Alteromonadaceae	312	–0.09090909	0.7832
	*Alteromonadaceae*_unclassified	212	0.2587413	0.4169
	Colwelliaceae	334	–0.1678322	0.6037
	Nitrincolaceae	372	–0.3006993	0.3425
	Pseudoalteromonadaceae	228	0.2027972	0.5281
	Saccharospirillaceae	212	0.2587413	0.4169
Deltaproteobacteria	*Desulfovibrionaceae*	194	0.3216783	0.3083
	Desulfobacteraceae	138	0.5174825	0.08865
	Desulfococcaceae	148	0.4825175	0.1154
Campilobacterota	*Arcobacteraceae*	42	0.8531469	**0.0007719****
	Rs-M59_termite_group	188	0.3426573	0.2762
Firmicutes	*Caminicellaceae*	170	0.4055944	0.1926
	Lachnospiraceae	154	0.4615385	0.1338
	*Lachnospirales*_unclassified	150	0.4755245	0.1213
	Clostridiaceae	172	0.3986014	0.201
Bacteroidota	*Flavobacteriaceae*	418	–0.4615385	0.1338
	Saprospiraceae	286	0	1
	*Bacteroidia*_unclassified	430	–0.5034965	0.09875
Deferribacterota	*Deferribacteraceae*	142	0.5034965	0.09875
Verrucomicrobiota	P.palmC41_fa	302	–0.05594406	0.869

*1 Significant of the *p* value are marked in bold with * $p < 0.05$ and ** $p < 0.01$.

Supplementary Table 4 Primers for bacterial community analysis and probes for FISH used in this study.

Analysis	Name	Sequence (5 '–3 ')	Targeted organism	*E.coli* position	Ref.
Bacterial community analysis	515F	GTGCCAGCMGCCGCGGTAA	Most bacteria	515	[15,16]
	806R	GGACTACHVGGGTWTCTAAT	Most bacteria	806	[15,16]
FISH	EUB338 mix	GCTGCCTCCCGTAGG AGT<br>GCAGCCACCCGTAGGTGT<br>GCTGCCACCCGTAGGTGT	Most bacteria	338	[17,18]
	Arc94	TGCGCCACTTAGCTGACA	*Arcobacteraceae*	94	[19]
	Non338	ACATCCTACGGGAGGC	Non-target	–	[20]

Supplementary Table 5 Raw reads of amplicon sequences in this study.

Sample ID *1	Number of read
AF_01	175,334
AF_02	128,384
AF_03	151,293
AF_04	126,044
AF_09	113,541
AF_10	134,315
SF_02	145,487
SF_04	133,751
SF_05	133,937
SF_06	139,962
SF_07	130,622
SF_08	143,521

*1 Sample ID indicate as ‘AF’ and ‘SF’ that collected from Aka Island and Sesoko Island, respectively.

Supplementary Figures

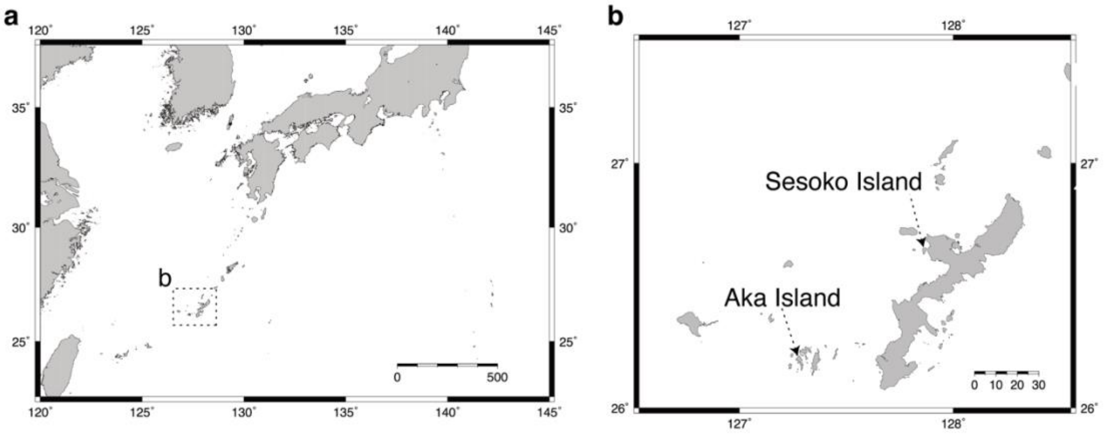

Supplementary Figure 1 Map showing the locations of two study sites in Okinawa, Japan (a-b). Sesoko Island and Aka Island are separated by more than 70 km (**b**).

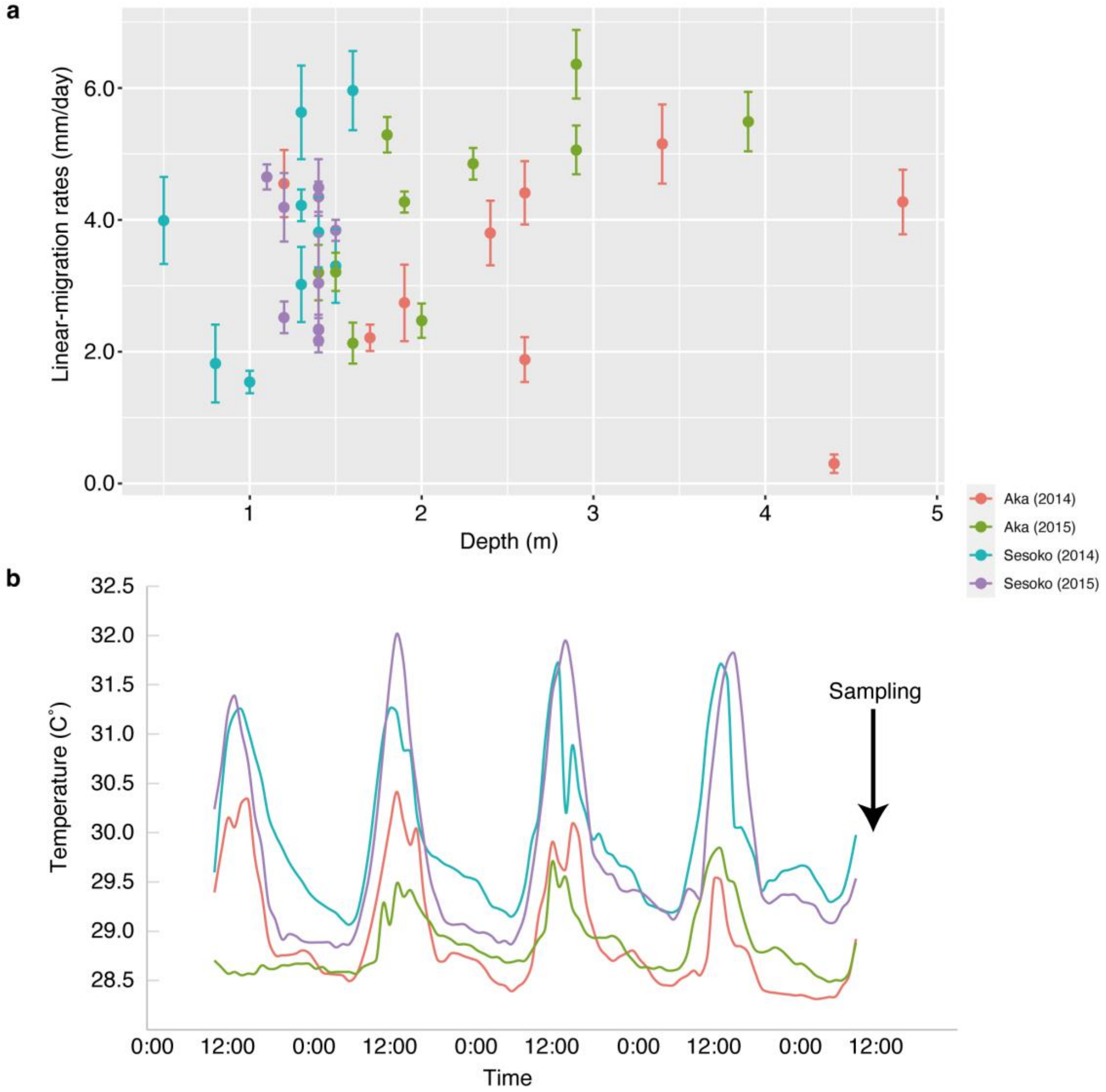

Supplementary Figure 2 Linear-migration rates of BBD and the depths (a) and water temperature prior to the sampling (from 4 days before, b) at Sesoko Island and Aka Island in 2014 and 2015. Sampling dates: 24th Aug. at Sesoko and 31st Aug. at Aka in 2014, and 3rd Aug. at Sesoko and 28th Aug. at Aka in 2015.

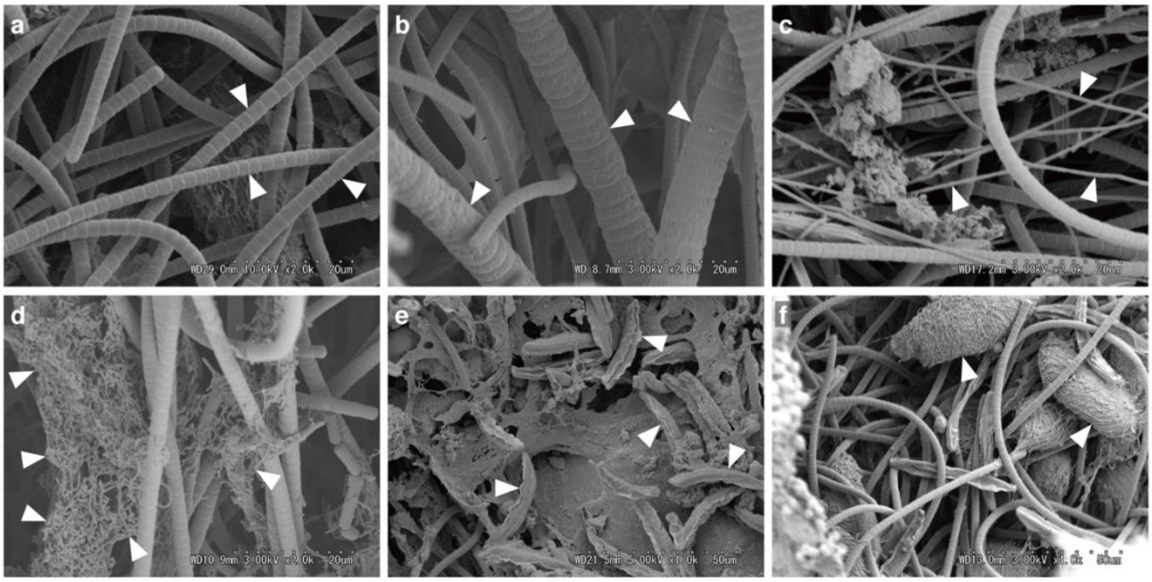

Supplementary Figure 3 Representative microbes on the BBD surface. SEM images of **a**) filamentous cyanobacteria (cell length: 2.69 ± 0.04 μm long and 2.11 ± 0.32 μm broad), **b**) thick cyanobacteria (2.63±0.16 μm long and 8.56±0.11 μm broad), **c**) filamentous microorganisms (0.76±0.32 μm broad**)**, **d**) bacterial aggregation, and **e-f**) two kinds of ciliates (Type A(**e**): 31.3±1.07 μm long and 5.53±0.18 μm broad, and Type B (**f**): 40.8±0.91 μm long and 17.5±0.51 μm broad). The scale bars indicate 20 µm (**a**-**d**) and 50 µm (**e**-**f**).

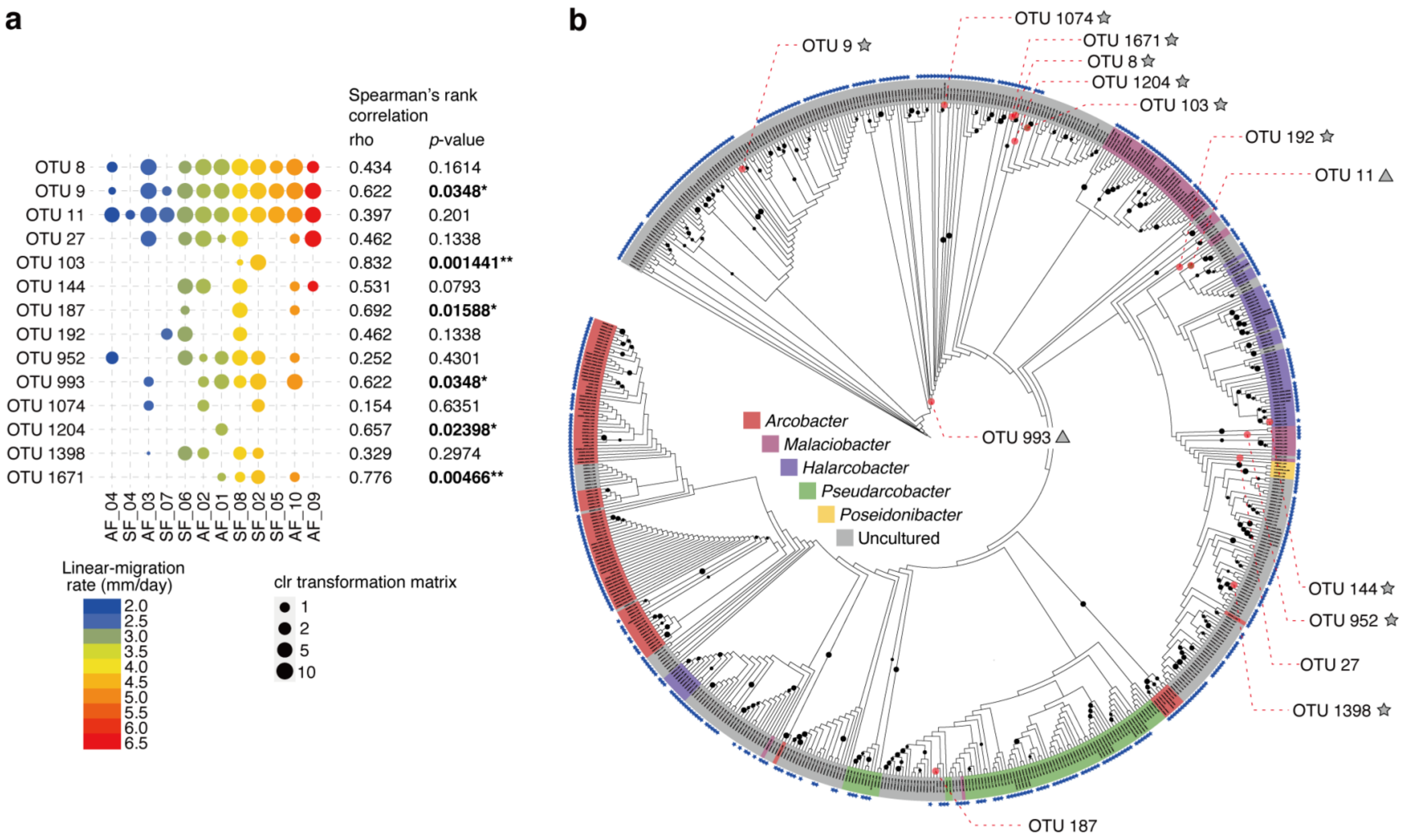
a
Spearman's rank correlation
rho
p-value
OTU 8 0.434 0.1614
OTU 9 0.622 0.0348*
OTU 11 0.397 0.201
OTU 27 0.462 0.1338
OTU 103 0.832 0.001441**
OTU 144 0.531 0.0793
OTU 187 0.692 0.01588*
OTU 192 0.462 0.1338
OTU 952 0.252 0.4301
OTU 993 0.622 0.0348*
OTU 1074 0.154 0.6351
OTU 1204 0.657 0.02398*
OTU 1398 0.329 0.2974
OTU 1671 0.776 0.00466**
AF_04 SF_04 AF_03 SF_07 SF_06 AF_02 AF_01 SF_08 SF_02 SF_05 AF_10 AF_09
Linear-migration rate (mm/day)
2.0 2.5 3.0 3.5 4.0 4.5 5.0 5.5 6.0 6.5
clr transformation matrix
1 2 5 10
b
OTU 9
OTU 1074
OTU 1671
OTU 8
OTU 1204
OTU 103
OTU 192
OTU 11
OTU 993
Arcobacter
Malaciobacter
Halarcobacter
Pseudarcobacter
Poseidonibacter
Uncultured
OTU 144
OTU 952
OTU 27
OTU 1398
OTU 187

Supplementary Figure 4 Partial correlation between clr transformation matrixes of each OTU in *Arcobacteraceae* and BBD-progressions (a) and phylogenetic placement of 14 OTU sequences on reference tree of family *Arcobacteraceae* (b). (**a**)The clr transformation matrixes of each OTU were calculated spearman's rank correlation with liner-progression rates and showed significant that are marked in bold with * $p < 0.05$ and ** $p < 0.01$ (**a**). The bubble chart showing clr transformation matrix (depicting by size) and progression rates (depicting by color). (**b**) The reference tree was generated from an alignment of 16S rRNA genes from a total of 653 sequences, which comprise five genera and uncultured group (**b**). The tree was constructed with the Maximum Likelihood method with 200 bootstrap replicates (only bootstrap values greater than 50% are denoted as black cycle at the node). For the outmost small bule star marks, it indicates that specific probe Arc94 can match the corresponding sequences (probe-matched 509 sequences). The OTUs labels with a grey star and a grey triangle on the tree indicate probe-match (a single node corresponding to a sequence that matched the probe is defined) and unclear (contains multiple nodes including miss-matched probe), respectively. The OTUs labeled without a symbol indicate that a single node is defined but mismatched with the probe.

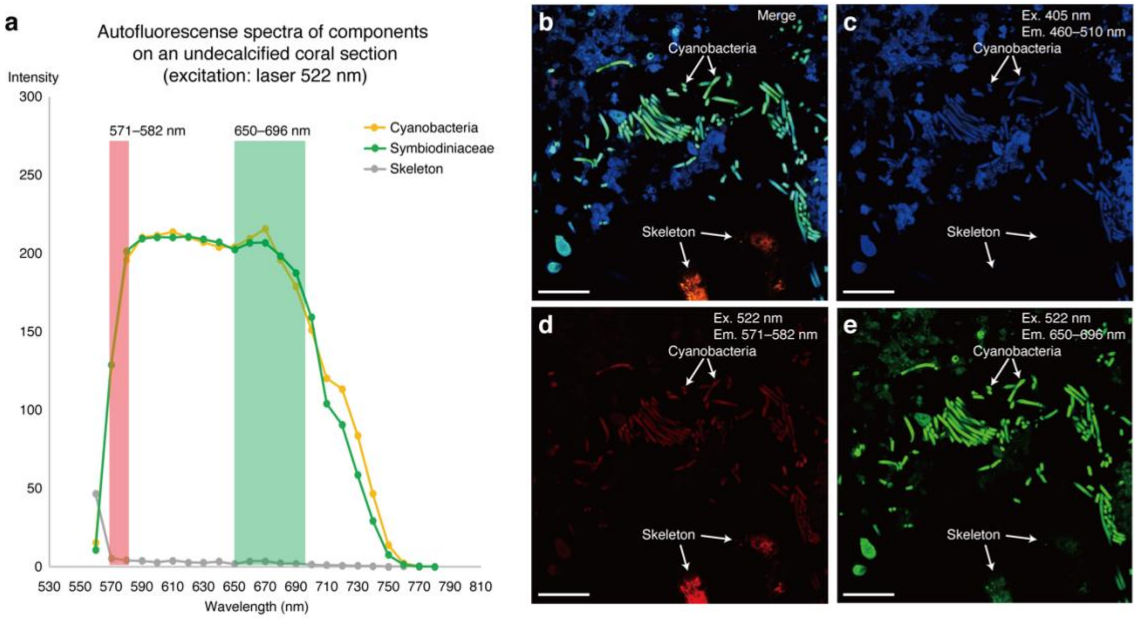

Supplementary Figure 5 Autofluorescence spectra at excitation of a laser 552 nm on an undecalcified coral section (a) and fluorescence *in situ* hybridization image using a negative control probe Non338 (b). Autofluorescence spectra (**a**) of cyanobacteria, Symbiodiniaceae and skeleton on a FISH-untreated undecalcified coral section, excited by a laser 522 nm (5% intensity), using a Lambda scan function of a confocal (TCS SP8, Leica). FISH images using the probe Non338 labeled with Cy3 showing a merged image (**b**) from three different channels (**c**-**e**). Given that skeleton is not shown autofluorescence, the FISH result indicating the non-specific binding on skeleton region from Non338 probe. Scale bars indicate 50 µm.

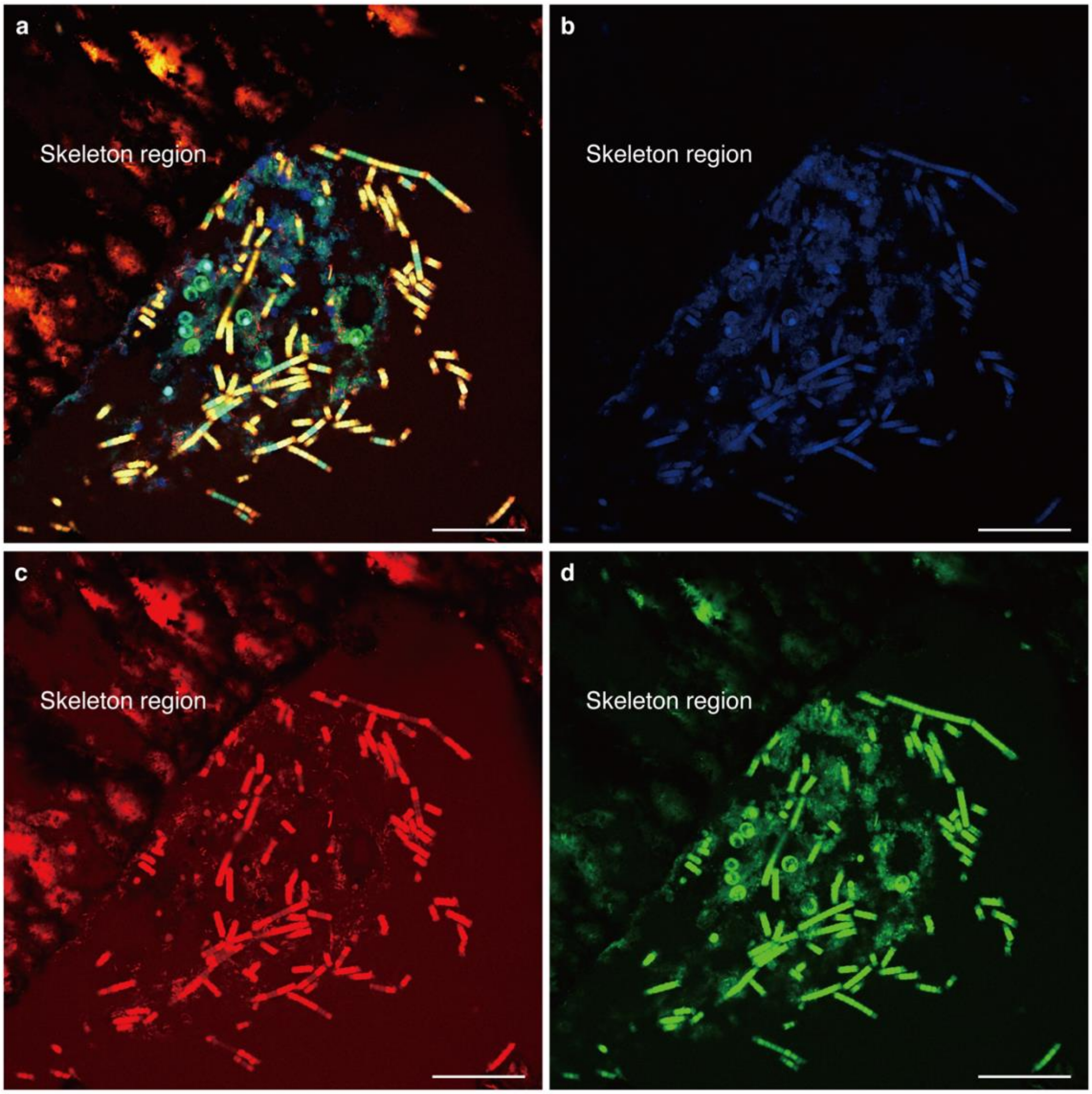

Supplementary Figure 6 Fluorescence *in situ* hybridization images showing EUB338mix probe (labeled with Cy3) binding with bacteria and cyanobacteria on an undecalcified coral section. Merged image (a) is displayed signals from mainly coral autofluorescence (**b**, blue) excited at leaser 405 nm (emission: 460–510 nm), Cy3 fluorochrome (**c**, red) excited at 552 nm (emission: 571–582 nm), and mainly chlorophyl (**d**, green) excited at 552 nm (emission: 650–696 nm). Scale bars indicate 50 µm.

Supplementary Note

Supplementary Note 1

At the family level, overall relative abundance (with top 24 families) of bacterial communities presented two families in *Cyanobacteria*: *Desertifilaceae, Oscillatoriaceae,* one family in *Alfaproteobacteria*: *Rhodobacteraceae*, seven families in *Gammaproteobacteria*: *Vibrionaceae, Alteromonadaceae, Alteromonadaceae*_unclassified, *Colwelliaceae, Nitrincolaceae, Pseudoalteromonadaceae, Saccharospirillaceae*, three families in *Deltaproteobacteria*: *Desulfovibrionaceae*, *Desulfobacteraceae*, *Desulfococcaceae,* two families in *Campilobacterota*: *Arcobacteraceae*, Rs-M59_termite_group, four families in *Firmicutes*: *Caminicellaceae, Lachnospiraceae, Lachnospirales*_unclassified, *Clostridiaceae*, three families in *Bacteroidota*: *Flavobacteriaceae*, *Saprospiraceae*, *Bacteroidia*_unclassified, one family in *Deferribacterota*: *Deferribacteraceae*, and one family in *Verrucomicrobiota*: P.palmC41_fa (**Fig. 3b** and **Supplementary Table 1**).

To confirm the presence of cyanobacteria, SRB, SOB (except for the *Rhodobacteraceae* and *Arcobacteraceae*), and other heterotrophic bacteria that indicate BBD-microbiome reported in previous studies, we focused on the representative OTUs (operational taxonomic unit[s]) that considering compromised >1% of total relative abundance (**Supplementary Table 2**). The most dominant bacterial family was the cyanobacterial family *Desertifilaceae,* which was currently proposed as new family in order *Oscillatoriales*[21], accounting for 42.57% of total relative abundance (**Supplementary Table 1**). In the *Desertifilaceae*, OTU 1 contributed predominantly or rarely to all microbial communities (in ranging from 1.38 to 77.67%) and affiliated with *Roseofilum* AO1-A which was isolated from BBD in Australia[2] and detected across the Indo-Pacific (**Supplementary Table 2**). As other dominant cyanobacteria affiliated with the family *Oscillatoriaceae*, OTU 23 was found in four samples from the both locations (in the ranging from 0.03 to 9.71%) with the linear-migration rates from 2.34 to 4.19 mm/day, and closely related with 98.01% similarity to *Vermifilum ionodolium* (accession No. MT321585.1) that retrieved from coastal limestone in USA. Interestingly, the second closest sequence (DQ446127.2) of OTU 23 according to 96.02% similarity were obtained from BBD in Bahamas (**Supplementary Table 2**).

SRB belonging to the *Desulfovibrionaceae* and *Desulfobacteraceae*, respectively represented by OTU 12 and OTU 5, showed ranged in proportions between 0.01 and 4.10% and between 0.02 and 7.98%. The OTU 12 affiliated to the *Desulfovibrionaceae* had a 100% similarity with an uncultured delta proteobacterium clone CD22D5 (AY497300.1) that was detected in BBD mat in the Caribbean Sea (**Supplementary Table 2**). The OTU 5 in *Desulfobacteraceae* was also closely related to uncultured bacterium Thai19_G02 (99.6% similarity), obtained from the coral disease white plague in Thailand, as the second closest lineage (**Supplementary Table 2**).

Besides the *Rhodobacteraceae* and *Arcobacteraceae,* we found only few reads of a common SOB member *Beggiatoa* spp. in our samples. In the family *Beggiatoaceae*, three OTUs (OTU 65, OTU 140 and OTU 815) accounted for the relative abundance ranging from 0.0018 to 0.98% in seven samples with the linear-migration rates from 2.13 to 4.85 mm/day (data not shown).

In other heterotrophic bacteria, the represented OTUs in most of families such as *Vibrionaceae, Alteromonadales*_unclassified, *Colwelliaceae, Nitrincolaceae, Pseudoalteromonadaceae, Caminicellaceae, Lachnospiraceae,* and *Lachnospirales*_unclassified showing varied relative abundances among our samples were linked with BBD and other coral diseases (**Supplementary Table 2**). Notably, OTU 7 in *Vibrionaceae* showed high relative abundance with 18.74% in samples with the fastest migration rates (**Fig. 3** and **Supplementary Table 2**). Although the amplicon sequences could only provide good resolution at the genus level[3], the best hit list of the sequences indeed included several *Vibrio* type strains in the blast search.

Supplementary References

1. Rasoulouniriana, D. *et al.* Pseudoscillatoria coralii gen. nov., sp. nov., a cyanobacterium associated with coral black band disease (BBD). *Dis. Aquat. Organ.* **87**, 91–96 (2009).
2. Buerger, P., Alvarez-Roa, C., Weynberg, K. D., Baekelandt, S. & van Oppen, M. J. H. Genetic, morphological and growth characterisation of a new *Roseofilum* strain (Oscillatoriales, Cyanobacteria) associated with coral black band disease. *PeerJ* **4**, e2110 (2016).
3. Hadaidi, G. *et al.* Ecological and molecular characterization of a coral black band disease outbreak in the Red Sea during a bleaching event. *PeerJ* **6**, (2018).
4. Hutabarat, P. U. B., Nguyen, X. H. & Suda, S. Black Band disease-related (BBD) cyanobacterium from Okinawan corals. *J. Appl. Phycol.* **30**, 3197–3203 (2018).
5. Berthold, D. E., Lefler, F. W. & Laughinghouse, H. D. Untangling filamentous marine cyanobacterial diversity from the coast of South Florida with the description of Vermifilaceae fam. nov. and three new genera: Leptochromothrix gen. nov., Ophiophycus gen. nov., and Vermifilum gen. nov. *Mol. Phylogenet. Evol.* **160**, 107010 (2021).
6. Sekar, R., Kaczmarsky, L. & Richardson, L. Microbial community composition of black band disease on the coral host Siderastrea siderea from three regions of the wider Caribbean. *Mar. Ecol. Prog. Ser.* **362**, 85–98 (2008).
7. Frias-Lopez, J., Klaus, J. S., Bonheyo, G. T. & Fouke, B. W. Bacterial community associated with black band disease in corals. *Appl. Environ. Microbiol.* **70**, 5955–5962 (2004).
8. Meron, D. *et al.* The impact of reduced pH on the microbial community of the coral Acropora eurystoma. *ISME J.* **5**, 51–60 (2011).
9. Roder, C. *et al.* Bacterial profiling of White Plague Disease in a comparative coral species framework. *ISME J.* **8**, 31–39 (2014).
10. Cooney, R. P. *et al.* Characterization of the bacterial consortium associated with black band disease in coral using molecular microbiological techniques. *Environ. Microbiol.* **4**, 401–413 (2002).
11. Klaus, J. S., Janse, I. & Fouke, B. W. Coral Black Band Disease Microbial Communities and

Genotypic Variability of the Dominant Cyanobacteria (CD1C11). *Bull. Mar. Sci.* **87**, 795–821 (2011).
12. Sunagawa, S. *et al.* Bacterial diversity and White Plague Disease-associated community changes in the Caribbean coral Montastraea faveolata. *ISME J.* **3**, 512–521 (2009).
13. Jones, R. J., Bowyer, J., Hoegh-Guldberg, O. & Blackall, L. L. Dynamics of a temperature-related coral disease outbreak. *Mar. Ecol. Prog. Ser.* **281**, 63–77 (2004).
14. Ben-Dov, E., Siboni, N., Shapiro, O. H., Arotsker, L. & Kushmaro, A. Substitution by Inosine at the 3′-Ultimate and Penultimate Positions of 16S rRNA Gene Universal Primers. *Microb. Ecol.* **61**, 1–6 (2011).
15. Apprill, A., McNally, S., Parsons, R. & Weber, L. Minor revision to V4 region SSU rRNA 806R gene primer greatly increases detection of SAR11 bacterioplankton. *Aquat. Microb. Ecol.* **75**, 129–137 (2015).
16. Walters, W. *et al.* Improved Bacterial 16S rRNA Gene (V4 and V4-5) and Fungal Internal Transcribed Spacer Marker Gene Primers for Microbial Community Surveys. *mSystems* **1**, e00009-15 (2016).
17. Daims, H., Brühl, A., Amann, R., Schleifer, K. H. & Wagner, M. The domain-specific probe EUB338 is insufficient for the detection of all Bacteria: development and evaluation of a more comprehensive probe set. *Syst. Appl. Microbiol.* **22**, 434–444 (1999).
18. Amann, R. I. *et al.* Combination of 16S rRNA-targeted oligonucleotide probes with flow cytometry for analyzing mixed microbial populations. *Appl. Environ. Microbiol.* **56**, 1919–1925 (1990).
19. Snaidr, J., Amann, R., Huber, I., Ludwig, W. & Schleifer, K. H. Phylogenetic analysis and in situ identification of bacteria in activated sludge. *Appl. Environ. Microbiol.* **63**, 2884–2896 (1997).
20. Wallner, G., Amann, R. & Beisker, W. Optimizing fluorescent in situ hybridization with rRNA-targeted oligonucleotide probes for flow cytometric identification of microorganisms. *Cytometry* **14**, 136–143 (1993).
21. Hašler, P., Casamatta, D., Dvořák, P. & Poulíčková, A. *Jacksonvillea apiculata* (Oscillatoriales, Cyanobacteria) *gen* . & *sp. nov* .: a new genus of filamentous, epipsamic cyanobacteria from North Florida. *Phycologia* **56**, 284–295 (2017).
22. Yu, J. *et al.* Species-specific Identification of Vibrio sp. based on 16S-23S rRNA gene internal transcribed spacer. *J. Appl. Microbiol.* **129**, 738–752 (2020).
