# Supplementary material for: Microbial mat compositions and localization patterns explain the virulence of black band disease in corals
Source: NPJ Biofilms Microbiomes. 2023 Apr 4;9:15. doi: 10.1038/s41522-023-00381-9 (PMC10073141; doi:10.1038/s41522-023-00381-9)
Supplement: Supplementary file 2 — Reporting Summary [file 41522_2023_381_MOESM2_ESM.pdf]

nature portfolio

Corresponding author(s): Sen-Lin Tang

Last updated by author(s): Jan 16, 2023

Reporting Summary

Nature Portfolio wishes to improve the reproducibility of the work that we publish. This form provides structure for consistency and transparency in reporting. For further information on Nature Portfolio policies, see our Editorial Policies and the Editorial Policy Checklist.

Statistics

For all statistical analyses, confirm that the following items are present in the figure legend, table legend, main text, or Methods section.

n/a	Confirmed	
☐	☒	The exact sample size (*n*) for each experimental group/condition, given as a discrete number and unit of measurement
☐	☒	A statement on whether measurements were taken from distinct samples or whether the same sample was measured repeatedly
☐	☒	The statistical test(s) used AND whether they are one- or two-sided<br>*Only common tests should be described solely by name; describe more complex techniques in the Methods section.*
☐	☒	A description of all covariates tested
☐	☒	A description of any assumptions or corrections, such as tests of normality and adjustment for multiple comparisons
☐	☒	A full description of the statistical parameters including central tendency (e.g. means) or other basic estimates (e.g. regression coefficient) AND variation (e.g. standard deviation) or associated estimates of uncertainty (e.g. confidence intervals)
☐	☒	For null hypothesis testing, the test statistic (e.g. *F*, *t*, *r*) with confidence intervals, effect sizes, degrees of freedom and *P* value noted<br>*Give P values as exact values whenever suitable.*
☒	☐	For Bayesian analysis, information on the choice of priors and Markov chain Monte Carlo settings
☐	☒	For hierarchical and complex designs, identification of the appropriate level for tests and full reporting of outcomes
☐	☒	Estimates of effect sizes (e.g. Cohen's *d*, Pearson's *r*), indicating how they were calculated

Our web collection on statistics for biologists contains articles on many of the points above.

Software and code

Policy information about availability of computer code

Data collection	Raw sequence data was preprocessed into Operational taxonomic Units (OTUs) at 97% nucleotide identity using MacQIIME, MOTHUR and USEARCH. For FISH experiment, area of pixel was extracted using software Fiji.
Data analysis	Statistics analyses were performed in R ver. 4.0.2 using following packages; phyloseq, vegan, microbiome.

For manuscripts utilizing custom algorithms or software that are central to the research but not yet described in published literature, software must be made available to editors and reviewers. We strongly encourage code deposition in a community repository (e.g. GitHub). See the Nature Portfolio guidelines for submitting code & software for further information.

Data

Policy information about availability of data

All manuscripts must include a data availability statement. This statement should provide the following information, where applicable:

- Accession codes, unique identifiers, or web links for publicly available datasets
- A description of any restrictions on data availability
- For clinical datasets or third party data, please ensure that the statement adheres to our policy

This data for this study is available through the DNA Data Bank of Japan (DDBJ) under DDBJ Read Archive: accession number DRA010783.

Human research participants

Policy information about studies involving human research participants and Sex and Gender in Research.

Reporting on sex and gender	n/a
Population characteristics	n/a
Recruitment	n/a
Ethics oversight	n/a

Note that full information on the approval of the study protocol must also be provided in the manuscript.

Field-specific reporting

Please select the one below that is the best fit for your research. If you are not sure, read the appropriate sections before making your selection.

☐ Life sciences ☐ Behavioural & social sciences ☒ Ecological, evolutionary & environmental sciences

For a reference copy of the document with all sections, see nature.com/documents/nr-reporting-summary-flat.pdf

Ecological, evolutionary & environmental sciences study design

All studies must disclose on these points even when the disclosure is negative.

Study description	To characterize the microbial consortium involved in the migration rate of coral Black Band Disease (BBD) and virulence, we examined whether the BBD-migration rate, as a proxy of the virulence, reflects the composition and locality of bacterial communities within the BBD mat
Research sample	Fragments of BBD-affecting coral Montipora were collected from two coral reefs in Okinawa, Japan.
Sampling strategy	Each sample (one sample per an individual colony) of the BBD region containing healthy tissue and skeleton (approximately 3-4 cm square and one cm depth) was cut using a hammer and a chisel at the area where the linear-migration rates were measured.
Data collection	Data were collected by SEM observation, 16S amplicon sequence and FISH experiment.
Timing and spatial scale	The linear migration rate was measured over a three days period before the sampling. Sampling dates: 24th Aug. at Sesoko and 31st Aug. at Aka in 2014, and 3rd Aug. at Sesoko and 28th Aug. at Aka in 2015.
Data exclusions	In bacterial community analysis, sequences from Eukaryota, Archaea, unknown and chloroplast were excluded.
Reproducibility	Raw data are shared via DNA Data Bank of Japan (DDBJ).
Randomization	BBD-affecting corals were measured the linear migration rates and taken randomly from the two reefs in 2014 and 2015.
Blinding	n/a

Did the study involve field work? ☒ Yes ☐ No

Field work, collection and transport

Field conditions	Samples were collected from two fringing reefs. The work was performed in the water depths shallower than 8 meters.
Location	Samples were collected from two reefs which located in Sesoko Island (26°38'35.2"N, 127°51'49.5"E) and Aka Island (26°12'00.0"N, 127°16'45.0"E) in Okinawa, Japan.
Access & import/export	After fixation, all samples were transported back to the lab from Okinawa under 4℃ and -80 ℃.
Disturbance	n/a

Reporting for specific materials, systems and methods

We require information from authors about some types of materials, experimental systems and methods used in many studies. Here, indicate whether each material, system or method listed is relevant to your study. If you are not sure if a list item applies to your research, read the appropriate section before selecting a response.

Materials & experimental systems

n/a	Involved in the study
☒	☐ Antibodies
☒	☐ Eukaryotic cell lines
☒	☐ Palaeontology and archaeology
☐	☒ Animals and other organisms
☒	☐ Clinical data
☒	☐ Dual use research of concern

Methods

n/a	Involved in the study
☒	☐ ChIP-seq
☒	☐ Flow cytometry
☒	☐ MRI-based neuroimaging

Animals and other research organisms

Policy information about studies involving animals; ARRIVE guidelines recommended for reporting animal research, and Sex and Gender in Research

Laboratory animals	n/a
Wild animals	Coral Montipora spp.
Reporting on sex	n/a
Field-collected samples	The sampling was conducted under prefectural government permits (Okinawa Prefectural Government permits; No. 26-46 for 2014 and No. 27-49 for 2015).
Ethics oversight	Okinawa Prefectural Government

Note that full information on the approval of the study protocol must also be provided in the manuscript.
